# Unraveling yield heterosis in Chinese cabbage hybrid by comparative transcriptomic analysis and *LHCB1* gene function analysis

**DOI:** 10.3389/fpls.2025.1627259

**Published:** 2025-07-02

**Authors:** Ruihua Wang, Min Han, Taili Han, Ligong Xu, Yuanyuan Li

**Affiliations:** ^1^ School of Advanced Agricultural Sciences, Weifang University, Weifang, China; ^2^ Vegetable Research Institute, Weifang Academy of Agricultural Sciences, Weifang, China

**Keywords:** Chinese cabbage hybrid, yield advantage, expression pattern, virus-induced gene silencing, weighted gene coexpression network

## Abstract

**Background:**

Changes in gene expression in plant hybrids are closely related to heterosis. Currently, few reports on key genes that promote yield advantage formation in Chinese cabbage hybrids exist.

**Methods:**

We conducted a comparative transcriptomic analysis between a Chinese cabbage hybrid (weichunbaiNo.3) and its parents using RNA sequencing, and the differentially expressed genes between the Chinese cabbage hybrid and its parents were confirmed in the rosette and the mature stages. The expression patterns of the differentially expressed genes were examined. The weighted gene coexpression network analysis and virus-induced gene silencing technology were employed to assess the key gene function in yield advantage formation of the Chinese cabbage hybrid.

**Results:**

In total, 3652 and 2768 genes were differentially expressed between the Chinese cabbage hybrid and its parents in the rosette and mature stages, respectively. These differentially expressed genes among the hybrid and its parents presented diverse expression patterns, and the expression levels of the most differentially expressed genes in the hybrid were higher than one of the parents but lower than another. The horticultural characteristics showed that weichunbai No. 3 hybrid had a greater yield advantage compared with parents. A vital hub gene related to yield, BraA09g035160.3C (an *LHCB1* gene), was identified by weighted gene coexpression network analysis. Through virus-induced gene silencing technology, the expression level of the BraA09g035160.3C gene in the hybrid was dramatically decreased, which slowed hybrid growth.

**Discussion:**

BraA09g035160.3C gene could play an important regulatory role in the yield advantage formation of weichunbai No. 3. These results will provide an important reference for in-depth research on the molecular mechanism underlying the yield advantage formation of Chinese cabbage hybrids.

## 1 Introduction

In plants, F_1_ hybrids show the superior performance relative to the parental lines, such as increased biomass, yield, and ability to resist stress (Hochholdinger and Hoecker, 2007). The excellent performance is attributed to changes in gene expression in hybrids. The genome modifications induced by the fusion of divergent genomes result in the remodeling of parental gene expression in hybrids, which is called “transcriptome shock” (Wu et al., 2016). The modes of gene action in hybrids encompass the levels of transcripts equal to the mid-parent (additivity), exceed the high parent (overdominance), fall below the low parent (underdominance), or align with either the high or low parent (high or low parent dominance) (Meyer et al., 2007). The additive expression balance of most genes in the hybrids provides a crucial foundation for the emergence of heterosis (Shi et al., 2011; Li et al., 2018). The additive gene expression has an influence on heterosis by balancing the expression of genes in metabolic pathways. The improved metabolic flux caused by additive gene expression brings about a more consistent, and lower variation in metabolites for maize hybrids compared with their inbred parents (Lisec et al., 2011). During late stages of development in maize hybrids, gene expression is shifted towards additive expression of the circadian-mediated carbon fixation and metabolic genes, contributing to biomass heterosis (Ko et al., 2016).

Genes showing statistically significant expression differences between the two groups are identified as differentially expressed genes (DEGs) (Lu et al., 2018). DEGs are usually closely related to the generation of hybrid vigor. The heterosis of K^+^ content in tobacco leaves was primarily driven by DEGs; The upregulation of genes involved in K^+^ uptake, transport, and root development enhanced K^+^ accumulation, leading to the heterotic effect (Mo et al., 2022). The high accumulation of secondary metabolites in tea hybrids was primarily attributed to DEGs involved in phenylpropanoid biosynthesis, flavonoid metabolism, cytochrome P450-mediated drug metabolism, and transcription factor regulatory pathways (Widhianata et al., 2022). DEGs between super hybrid rice varieties and their parents were significantly enriched in pathways related to yield and resistance, like circadian rhythm, photosynthetic process, and response to water deprivation (Fu et al., 2022). DEGs detected in the *Brassica rapa* F1 and its parents were primarily associated with auxin response, plant hormone signaling, purine metabolism, as well as starch and sucrose metabolism, which suggested that these biological processes may play a significant role in the heterosis of *Brassica rapa* (Liu et al., 2024).

Some DEGs are involved in the regulation of photosynthesis. Photosynthetic capacity is important for heterosis, and changes in the expression of photosynthesis-related genes directly affect photosynthesis efficiency, thereby affecting the plant growth and yield. The first step in the photosynthesis process is the light reaction occurring in thylakoid membranes (Kaiser et al., 2015). The light reaction process is completed by the photosystem I complex (PSI), cytochrome b6f protein complex (Cytb6f), photosystem II complex (PSII) and ATP synthase embedded in the thylakoid membranes (van Bezouwen et al., 2017). The knockdown of the *PsaF* gene (encoding the PSI-F subunit of photosystem I) prevents the light-harvesting complex I-730 from transferring energy to the P700 reaction center and leads to disorganization of the thylakoids, with the mutants exhibiting severe growth defects in *Arabidopsis thaliana* (Haldrup et al., 2000). Knockout lines of *PetM* (encoding cytochrome b6f complex subunit 7) have a decreased chloroplastic electron transport rate and decreased carotenoid and chlorophyll contents, resulting in decreased fruit yield in tomato (Bulut et al., 2023). In *Arabidopsis thaliana*, the absence of *LHCB1* (encoding the component of LHCII associated with PS II) results in chlorophyll loss, a pale green phenotype and growth delay; the absence of *LHCB1* also reduces the number of membrane layers per granum stack and the grana width (Vayghan et al., 2022). The lack of *AtCGL160* (required for the CF1-CFO assembly of ATP synthase) results in thylakoid deficiency in the chloroplast, coupled with the emergence of plastoglobuli in densely packed stromal clusters; in addition, catabolic activity is increased, and degradation processes are accelerated; consequently, photosynthetic efficiency is decreased, and plant growth is impaired (Reiter et al., 2023). In summary, the lack of genes related to photosynthetic reactions can reduce photosynthesis efficiency and have negative effects on plant growth and yield.

The gene silencing technique can be used to verify the biological functions of genes at the transcriptional or post-transcriptional level, such CRISPR/Cas9 and VIGS. CRISPR/Cas9 is used to verify the biological functions of genes at the transcriptional level, while the VIGS is performed at the post-transcriptional level to verify the biological functions of genes. Compared to CRISPR/Cas9, VIGS enables rapid transient gene silencing without genetic transformation, and it features a short experimental cycle and low cost. Various viruses have been modified as VIGS vectors to silence endogenous plant genes, such as potato virus X (PVX), tobacco rattle virus (TRV), turnip yellow mosaic virus (TYMV), and cabbage leaf curl virus (CaLCuV) (Faivre-Rampant et al., 2004; Burch-Smith et al., 2006; Pflieger et al., 2008; Xiao et al., 2020). CaLCuV belongs to the Begomovirus genus of the Biviridae family and has a wide host range. This virus is composed of two single-stranded circular DNAs: DNA-A and DNA-B. DNA-A encodes coat proteins, replication enhancer proteins, replication-associated proteins, putative pathogenesis-related proteins, and transcriptional activators, whereas DNA-B encodes two movement proteins; CaLCuV DNA-A and CaLCuV DNA-B are transformed into the PCVA and PCVB vectors, respectively (Tang et al., 2013). The PCVA/PCVB-based VIGS system has been used to successfully silence plant endogenous genes. The phytoene desaturase (*PDS*) gene encodes a key enzyme involved in carotenoid biosynthesis, and carotenoids can protect chlorophyll from light damage during photosynthesis. The Mg-chelatase H subunit (*ChlH*) gene (encoding a subunit of Mg-chelatase) is related to chlorophyll biosynthesis. Through PCVA/PCVB-mediated VIGS, the *PDS* and *ChlH* genes are silenced, leading to white and yellow leaf phenotypes, respectively (Xiao et al., 2020). The *BrERF109* gene highly induced by multiple abiotic stresses positively controls tolerance to salt and drought stresses; *BrERF109* is silenced by PCVA/PCVB-mediated VIGS, which results in the susceptibility of Chinese cabbage to salt and drought stresses, and superoxide dismutase and peroxidase activities are also suppressed (Li et al., 2024).

Chinese cabbage (*Brassica rapa* L. ssp. *pekinensis*), which belongs to the *Brassica* genus of Cruciferae, is rich in vitamins, dietary fiber and antioxidants and is an economically important vegetable crop. The main method of Chinese cabbage breeding is to take advantage of hybrid vigor. New varieties of Chinese cabbage with heterosis obtained by interspecific hybridization have high yields, good quality and strong resistance (Saurabh et al., 2019). The *Brassica rapa* L. ssp. *pekinensis* var. weichunbai No. 3 produced by hybridization has high levels of heterosis in yield and quality (Han et al., 2018). To understand the molecular mechanism of yield advantage formation in weichunbai No. 3, DEGs between weichunbai No. 3 and its parents were investigated. Hub genes were identified by weighted gene coexpression network analysis of the DEGs. The biological function of the vital gene was analyzed and verified using a PCVA/PCVB-based VIGS analysis. This study provides a comprehensive perspective for the study of the molecular mechanism of heterosis formation in Chinese cabbage hybrids.

## 2 Materials and methods

### 2.1 Plant materials

*Brassica rapa* L. ssp. *pekinensis* var. weichunbai No. 3 is a spring-heading Chinese cabbage variety suitable for planting in spring and has an obvious yield advantage (Han et al., 2018). Weichunbai No. 3 is an F_1_ hybrid bred by crossing the self-incompatible lines *Brassica rapa* L. ssp. *pekinensis* var. BZ07–09 as the maternal parent and *Brassica rapa* L. ssp. *pekinensis* var. BD05–272 as the paternal parent. These materials were provided by the Vegetable Research Institute at the Weifang Academy of Agricultural Science and were grown in the experimental fields of the Weifang Academy of Agricultural Sciences under natural conditions. The experimental materials were sown in March (spring). The outer leaf tissues of the rosette stage were collected in April (spring), and the outer leaf tissues of the mature stage were collected in May (spring). Each material had three biological replicates.

In the mature stage, each of the hybrid and its parents had twenty plants to be measured the seven horticultural traits. The seven horticultural traits were as follows: plant height, maximum leaf length, maximum leaf width, nonwrapper leaf (outer leaf) number, leaf number of the leafy head, leafy head height, leafy head weight, and plant weight. In addition, mid-parent heterosis (MPH) was calculated according to the **Equation 1**, and high-parent heterosis (HPH) was calculated according to the **Equation 2** (Shalby et al., 2021).


(1) $$MPH\left(\%\right)=\left(M_{HY}-M_{MiP}\right)/M_{MiP}*100$$

(2) $$HPH\left(\%\right)=\left(M_{HY}-M_{HiP}\right)/M_{HiP}*100$$

M_HY_ was the value of the hybrid; M_MiP_ was the average value of the two parents; M_HiP_ was the highest value of the two parents. M_HY_ = $\bar{X}$ _Z_, M_MiP_ = ( $\bar{X}$ _M_+ $\bar{X}$ _P_)/2, M_HiP_ = $\bar{X}$ _M_ or $\bar{X}$ _P_ ( $\bar{X}$ represented the average value of the trait values of twenty plants, Z represented the hybrid, M represented the maternal parent, P represented the paternal parent).

### 2.2 Library construction and sequencing

In the rosette stage, each material had three biological replicates, resulting in a total of nine samples. Similarly, in the mature stage, the maternal parent, paternal parent, and the hybrid also each had three biological replicates, leading to another total of nine samples. The total RNA of eighteen samples was extracted from leaf tissues using a TRIzol reagent kit according to the manufacturer’s protocol. The RNA quality was assessed and checked by Agilent 2100 Bioanalyzer, NanoPhotometer spectrophotometer, and RNase free agarose gel electrophoresis. Eighteen libraries of RNA-seq (each sample corresponding to a library) were constructed using the NEBNext^®^ RNA Library Prep Kit (NEB # E7530) following the manufacturer’s instructions. All cDNA libraries were checked by the Agilent 2100 bioanalyzer, NanoPhotometer spectrophotometer, and Qubit2.0 Fluorometer. After passing the quality inspection, the eighteen libraries were sequenced on the Illumina sequencing platform (Illumina HiSeq™4000) using 150 bp paired-end sequencing by Gene Denovo Biotechnology Co. (Guangzhou, China).

### 2.3 RNA-seq data analysis

Reads obtained from the sequencing machines included raw reads with adapters or low-quality bases, which affected the subsequent assembly and analysis. To obtain high-quality clean reads, reads were further filtered according to the following parameters: 1) reads containing adapters were removed; 2) reads containing more than 10% unknown nucleotides (N) were removed; and 3) low-quality reads containing more than 50% low-quality (Q value ≤ 20) bases were removed. Paired-end clean reads were mapped to the *B. rapa* v3.0 reference genome using HISAT2 (version 2.4) (Kim et al., 2015), and then the mapped reads were assembled into the transcripts using String Tie software (version 1.3.1) (Pertea et al., 2015). The levels of transcription were quantified by calculating fragments per kilobase of transcript per million fragments (FPKM) using RSEM (version 1.3.3) (Li and Dewey, 2011).

If the FPKM value of a gene was less than 1 in all samples, and it was considered a nonexpressed gene. Differential expression analysis between the hybrid and its parents was performed using DESeq2 software (version 1.2.10) (Love et al., 2014). The genes with the parameter of the absolute value of log2 fold change ≥1 and false discovery rate (FDR)< 0.05 were considered as differentially expressed genes (DEGs).

### 2.4 WGCNA

The DEGs and the trait data of eighteen sequenced samples were used to construct a gene coexpression network and analyze the correlation of modules and trait data by the WGCNA R (version 4.0.3) package. First, the soft threshold power (the weighting coefficient β) was selected by the pickSoftThreshold function. Second, the correlation matrix was obtained based on the Pearson’s correlations between all pairwise genes, and then the adjacency matrix was constructed according to the obtained correlation matrix using the selected weighting coefficient β. Third, the weighted adjacency matrix was transformed to a topological overlap matrix (TOM). Finally, genes with similar expression patterns were grouped into gene modules by running average linkage hierarchical clustering according to the TOM-based dissimilarity. Eventually, gene modules with different colors were identified by the dynamic tree cut algorithm (minimum cluster size of 30, merging threshold function of 0.25) (Yu et al., 2020).

Module eigengenes (MEs) of each gene module were estimated by principal component analysis (PCA) and defined as the first principal component, and the expression of MEs was considered representative of all genes in the corresponding module. In the rosette and mature stages, three growth characteristics (plant height-ZG, plant weight-DZG, and total leaf number-YPS) were measured for the hybrid and its parents. The Pearson correlation coefficient was calculated between each characteristic and the MEs of each module. P< 0.05 was regarded as a significant correlation between the characteristic and the module. For each module, module membership (MM) was calculated as the correlation between each gene in the module and its MEs. Genes with high MM values were highly connected to the corresponding module and had the high intramodular connectivity. In addition, gene significance (GS) was calculated by Pearson correlation analysis to reflect the correlation between growth characteristics and the expression levels of genes in the modules.

### 2.5 Virus-induced gene silencing experiment

The seeds of *Brassica rapa* L. ssp. *pekinensis* var. weichunbai No. 3 were sown in nutrient soil in a climate chamber at 16 h/8 h (day/night) and 23°C/19°C (day/night). The cabbage leaf curl virus (CaLCuV)-mediated VIGS system was applied to silence the target gene. CaLCuV gene-silencing vectors (PCVA/PCVB) were used to construct a recombinant gene expression silencing vector. In accordance with the reference sequence provided by the Brassicaceae Database (http://www.brassicadb.cn/#/), the primers with adapters were designed to insert the full-length CDS into the reconstructed PUC19 vector using a ClonExpress^®^ Ultra One Step Cloning Kit (Vazyme). The full-length CDS obtained by DNA sequencing was used to design the VIGS fragment. The 500 bp VIGS fragment was designed using the online SGN VIGS Tool (http://vigs.solgenomics.net/), and the adapter sequence was subsequently ligated to this fragment such that this fragment was inserted into the PCVA vector using the KpnI and XbaI restriction loci. The phytoene desaturase gene (*PDS*) was used to determine the effectiveness of the PCVA/PCVB-VIGS system. Plants at the four-true-leaf stage were used for infiltration. Sixty plants with consistent growth were selected and equally divided into four groups. The plants infiltrated with PCVA-PDS served as positive control. Noninfiltrated plants served as the No. 1 control group. The plants infiltrated by the empty vector PCVA constituted the No. 2 control group. The plants infiltrated with PCVA-target gene 500 bp served as the experimental group. The infiltration method used was the method described by Xiao et al. (2020). QRT-PCR was used to examine the expression levels of the target gene in the control and experimental groups.

## 3 Result

### 3.1 Yield heterosis in the weichunbai No. 3 hybrid

In the mature stage, weichunbai No. 3 and its parents each had twenty plants, which were used to measure horticultural traits. The average values of the seven horticultural characteristics of the twenty plants for the hybrid and its parents were shown in **Table 1**. The MPH values of all seven traits were positive, which demonstrated improvements compared with the average of the parents. The MPH value of plant weight was the highest, followed by that of leafy head weight and leaf number of the leafy head. The HPH values of all seven characteristics were also positive, indicating improvements over the highest value of the two parents. The HPH values of the plant weight and leafy head weight were the highest, followed by that of the leaf number of the leafy head. Therefore, the increases in leafy head weight and plant weight were the most prominent, with MPH and HPH values ≥100% (**Table 1**), which suggested a greater yield of the weichunbai No. 3 hybrid.

**Table 1:** Different horticultural traits of the Chinese cabbage hybrid and its parents.

	Plant Height (cm)	Maximum Leaf Length (cm)	Maximum Leaf Width (cm)	Non-Wrapper Leaf Number (piece)	Leaf Number of the Leafy Head (piece)	Leafy Head Weight (kg)	Plant Weight (kg)
weichunbai No. 3	42	44	29	15	65	3.2	4.8
BZ07-09	35	34	21	13	46	1.6	2.4
BD05-272	33	32	19	13	41	1.5	2.1
MPH	24%	33%	45%	15%	49%	106%	113%
HPH	20%	29%	38%	15%	41%	100%	100%

The values in the table represented the average values of different horticultural traits of the Chinese cabbage hybrid and its parents.

### 3.2 Analysis of DEGs in two developmental stages

**Figure 1:**
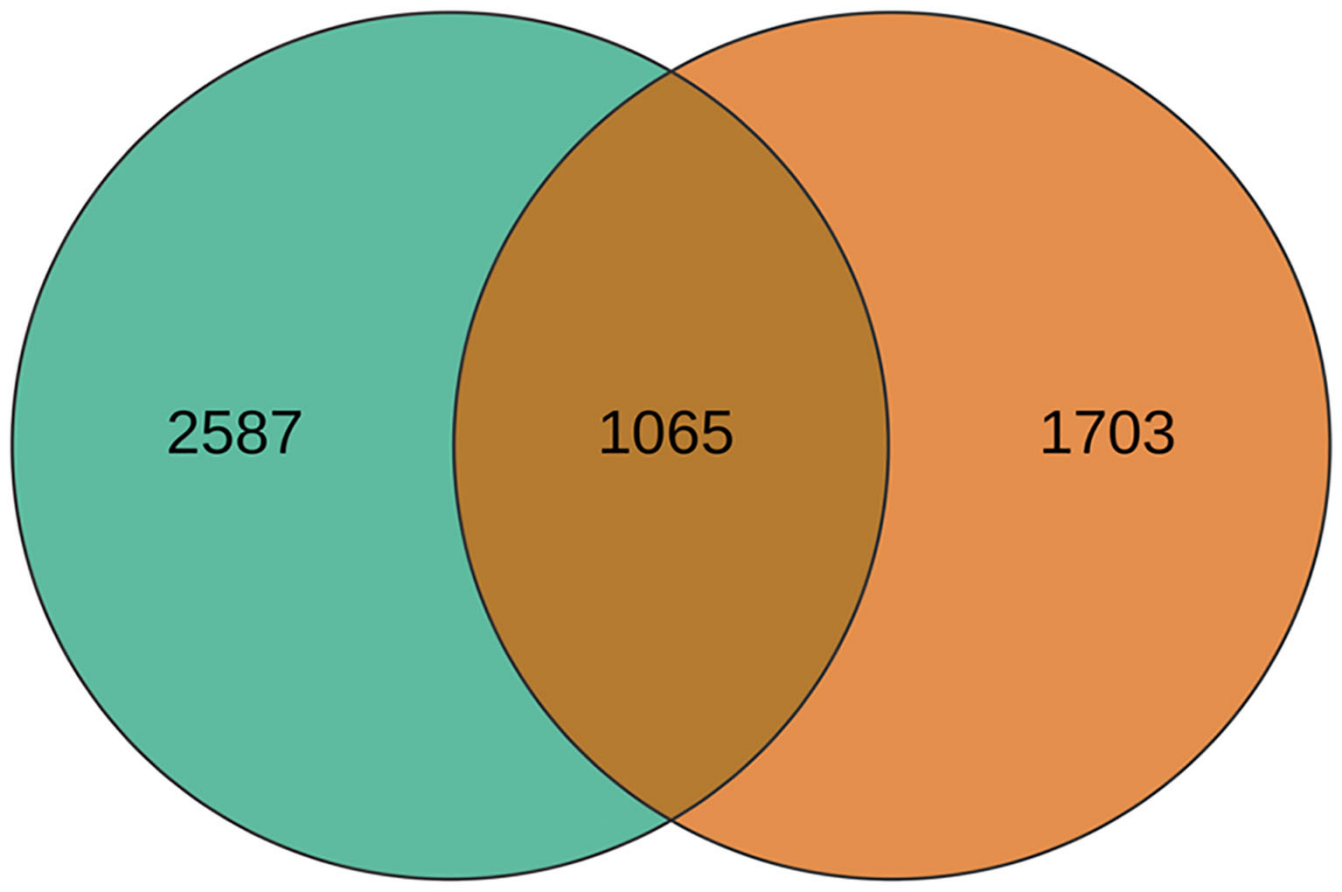
Venn diagram of DEGs between the hybrid and its parents in the two stages.

**Figure 2:**
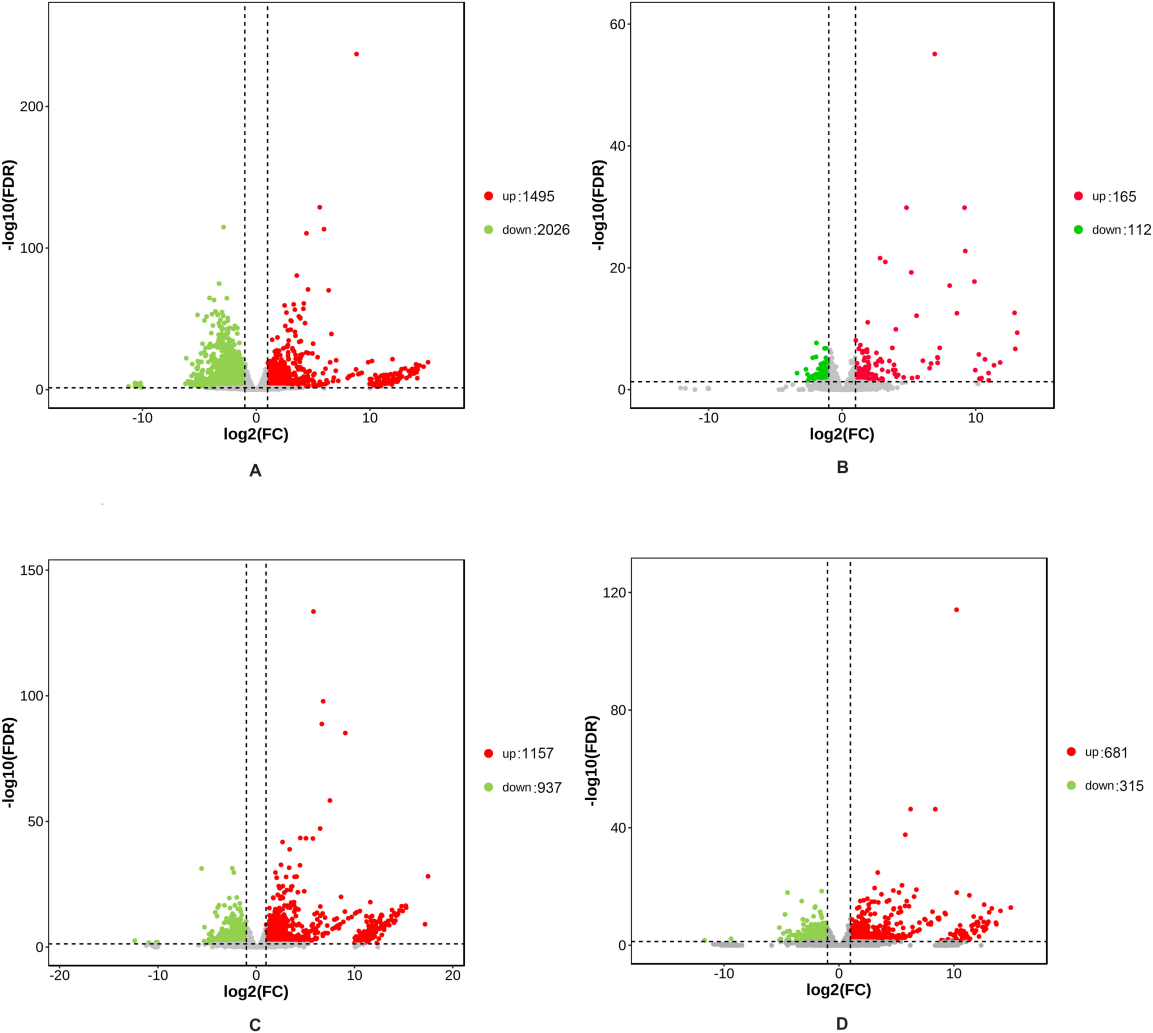
Volcano plots showing DEGs between the hybrid and its parents. The red plot represents the upregulated genes; the green plot represents the downregulated genes; and the gray plot represents the genes whose expression levels were not significantly different. **(A, B)** DEGs between the hybrid and its parents in the rosette stage. **(C, D)** DEGs between the hybrid and its parents in the mature stage. Log2 (FC) means that log2 transformation to fold changes of DEG expression between the hybrid and its parents. Log10 (FDR) means that log10 transformation to false discovery rate.

A total of 5355 DEGs were identified between the hybrid and its parents in two developmental stages (**Supplementary Table S1**), of which 1065 DEGs were common to both stages (**Figure 1**). In the rosette stage, there were 3652 differentially expressed genes (DEGs) between the hybrid and its parents, which included 3521 DEGs between the hybrid and the paternal parent (**Figure 2A**) and 277 DEGs between the hybrid and the maternal parent (**Figure 2B**). Obviously, the hybrid had a higher number of DEGs when compared with the paternal parent than when compared with the maternal parent. This situation also occurred in the mature stage, in which 2768 DEGs were identified between the hybrid and its parents. There were 2094 DEGs between the hybrid and the paternal parent (**Figure 2C**), whereas there were only 996 DEGs between the hybrid and the maternal parent (**Figure 2D**).

From the above results, it could be observed that the number of DEGs between the hybrid and parents was greater in the rosette stage than in the mature stage. The rosette stage was a critical period for the establishment of the photosynthetic system in Chinese cabbage, during which the rapid expansion of leaves required the coordinated expression of numerous metabolism-related genes (Sun et al., 2021). Heterosis was most pronounced during the biomass accumulation phase of the rosette stage in Chinese cabbage, necessitating the involvement of more differential genes in regulation. In addition, in both stages, the number of DEGs between the hybrid and paternal parent was greater than that between the hybrid and maternal parent. This lower expression divergence between the hybrid and the maternal parent may suggest a closer genetic association between the hybrid and the maternal parent than between the hybrid and the paternal parent. The genes in the hybrid presented expression levels with a bias to the maternal parent.

**Figure 3:**
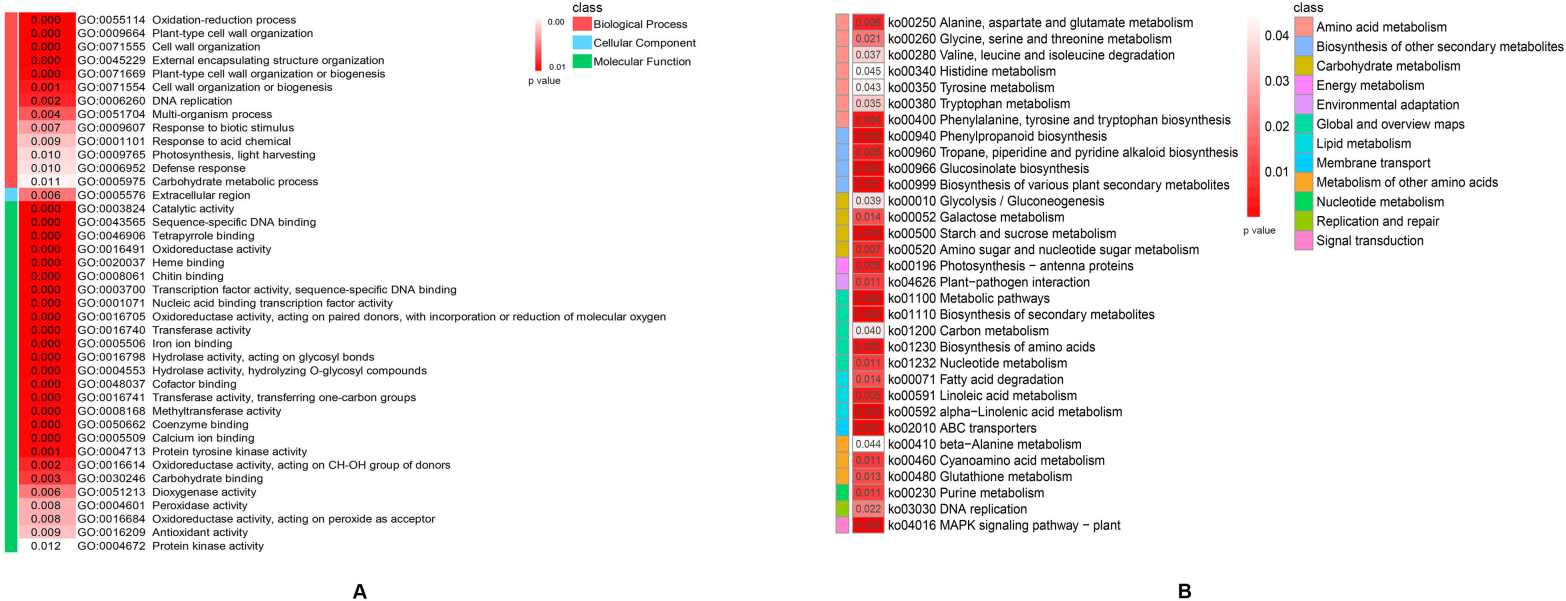
**(A)** The top 40 significantly enriched GO terms. **(B)** The significantly enriched KEGG metabolism pathways.

A total of 5355 DEGs between the hybrid and its parents in the two stages were subjected to Gene Ontology (GO) and Kyoto Encyclopedia of Genes and Genomes (KEGG) enrichment analyses, which contributed to a better understanding of the biological functions of the DEGs. DEGs were significantly enriched in 66 GO functional groups (p< 0.05), including 18 GO terms in the biological process group, 1 GO term in the cellular component group and 47 GO terms in the molecular function group (**Supplementary Table S2**). The top 40 significantly enriched GO terms were shown in **Figure 3A**. One significantly enriched GO term related to photosynthesis was ‘GO:0009765 photosynthesis, light harvesting’. KEGG pathway enrichment analysis was subsequently performed to identify heterosis-related metabolic pathways. The results revealed that 32 KEGG pathways were significantly enriched (p< 0.05, **Figure 3B**; **Supplementary Table S3**). ‘Lipid metabolism’, ‘carbohydrate metabolism’ and ‘energy metabolism’ could have vital effects on heterosis. Some DEGs whose expression levels are significantly greater in a cotton hybrid are enriched in lipid metabolism, carbohydrate metabolism, and photosynthesis processes, and increased lipid metabolism, carbohydrate metabolism, and photosynthesis capabilities contribute to heterosis formation at the seedling stage (Ding et al., 2021). The DEGs between a rice hybrid and its parents are enriched in energy metabolism, which is correlated with yield-related quantitative trait loci and plays an important role in the heterosis development (Wei et al., 2009). As shown in **Figure 3B**, ‘lipid metabolism’ included three metabolic pathways, ‘carbohydrate metabolism’ included four metabolic pathways, and ‘energy metabolism’ included only one metabolic pathway, ‘photosynthesis - antenna proteins’. In summary, DEGs enriched in these metabolic pathways could change the efficiency of the related metabolic pathways, which played a positive role in the hybrid vigor formation of weichunbai No. 3.

### 3.3 Identification of the expression patterns of DEGs

**Figure 4:**
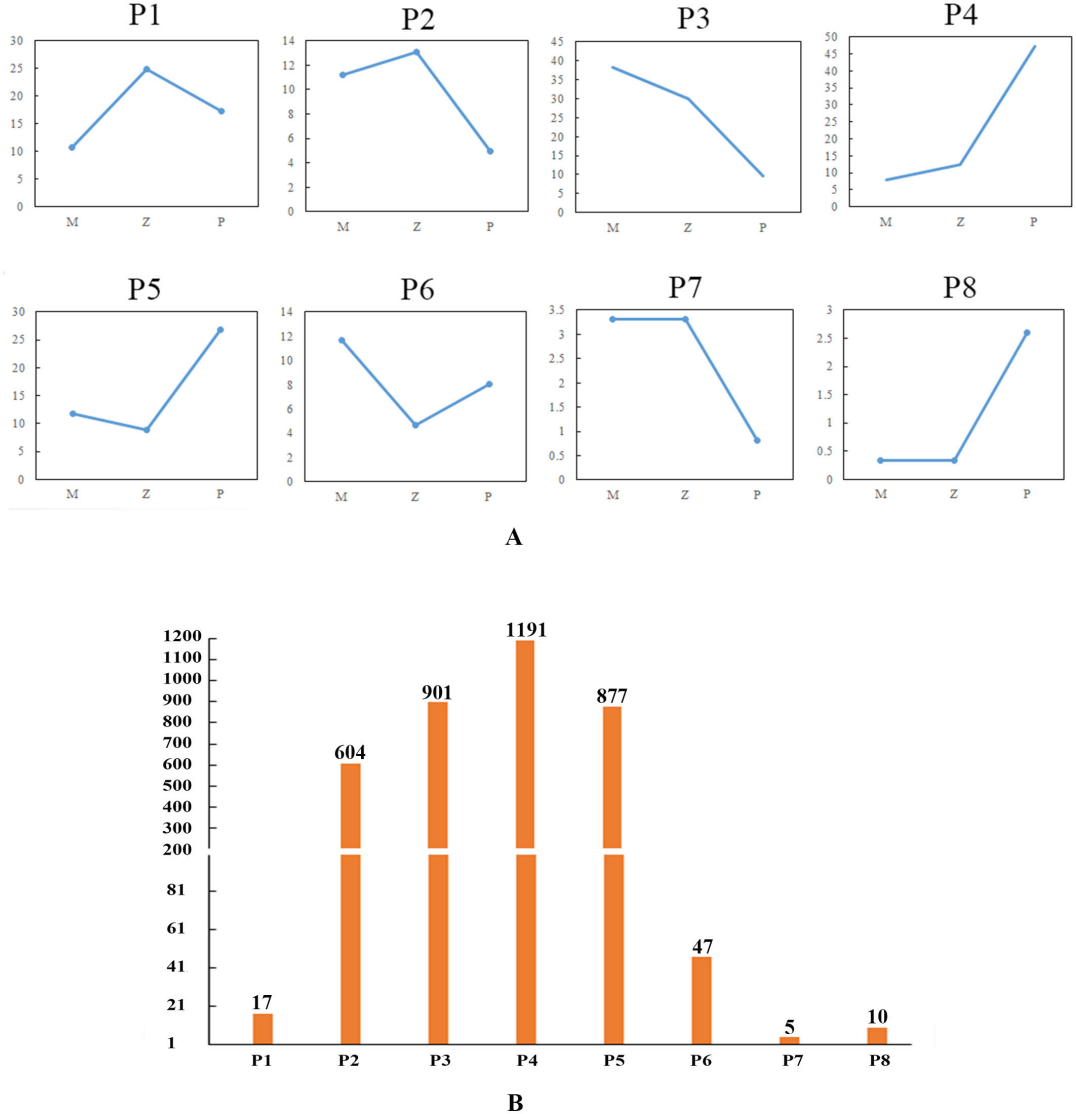
DEG expression patterns in the rosette stage. **(A)** Eight expression patterns. The ordinate is the average of all gene expression levels of the sample in the pattern, and the abscissa is the sample name; M represents the maternal parent; Z represents the hybrid; and P represents the paternal parent. **(B)** Number of DEGs associated with each pattern.

In the rosette stage, based on the expression levels of DEGs among the hybrid (Z), maternal parent (M), and paternal parent (P), the expression patterns of DEGs were divided into eight distinct types: Z>P>M (P1), Z>M>P (P2), M>Z>P (P3), P>Z>M (P4), P>M>Z (P5), M>P>Z (P6), M=Z>P (P7), and M=Z<P (P8) (**Supplementary Table S4**; **Figure 4**). P4 had the greatest number of DEGs, whereas P7 had the least number of DEGs. These patterns were divided into four groups: highest expression in the hybrid, lowest expression in the hybrid, expression of the hybrid between the parents, and expression of the hybrid equal to one of the parents. P1 and P2 showed that the expression levels of DEGs were the highest in the hybrid, and the number of genes in P2 (16.54%) was greater than that in P1 (0.47%). In P3 and P4, the expression levels in the hybrid were between those of the two parents, accounting for 57.28% of the DEGs. P5 and P6 presented that the expression levels of DEGs were the lowest in the hybrid, and the number of DEGs in P5 (24.01%) was greater than that in P6 (1.29%); moreover, the quantity difference was marked. In P7 and P8, the expression levels of the DEGs in the hybrid were equal to those in the maternal parent, and the number of genes associated with these two patterns was very low, accounting for only 0.41% of the total number of DEGs.

**Figure 5:**
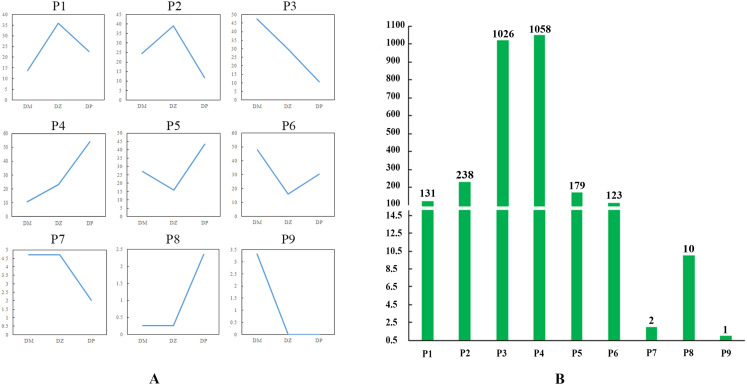
DEG expression patterns in the mature stage. **(A)** Nine expression patterns. The ordinate represents the average of all gene expression levels of the sample in the pattern, and the abscissa represents the sample name; DM represents the maternal parent; DZ represents the hybrid; and DP represents the paternal parent. **(B)** Number of DEGs associated with each pattern.

In the mature stage, based on the expression levels of DEGs among the hybrid (DZ), maternal parent (DM), and paternal parent (DP), the expression patterns of DEGs were divided into nine distinct types: Z>P>M (P1), Z>M>P (P2), M>Z>P (P3), P>Z>M (P4), P>M>Z (P5), M>P>Z (P6), M=Z>P (P7), M=Z<P (P8), and P=Z<M (P9) (**Supplementary Table S5**; **Figure 5**). P9, which contained only one DEG, presented a new pattern, in which the expression level of the hybrid was equal to that of the paternal parent. **Figure 5** exhibited that P4 had the greatest number of DEGs, whereas P9 had the least number of DEGs. Similar to the situation in the rosette stage, P2 (8.60%) had more DEGs than did P1 (4.73%), but the difference between them was smaller than that in the rosette stage. The number of DEGs in P3 and P4 accounted for 75.29% of the total DEGs, which was greater than that in the rosette stage. Although the number of DEGs in P5 (6.47%) was greater than that in P6 (4.44%), there was no significant difference in quantity between them, which was different from the rosette stage. The number of DEGs in P7 and P8 was still very low, accounting for 0.43% of the total DEGs.

DEGs in P3 and P4 patterns account for 57.28% and 75.29% in the rosette stage and mature stage, respectively, accounting for the majority of the total DEGs. Further analysis revealed that the DEGs in the P3 and P4 patterns of the two stages were all additively expressed genes. The additively expressed genes in the hybrid were identified with no significantly differential expression levels against mid-parent value (MPV) (Cheng et al., 2015). Researches indicated that the additive expression of genes in hybrids was closely related to heterosis formation (Guo et al., 2006; Thiemann et al., 2014; Dan et al., 2015; Liu et al., 2018). The additive expression could play an unusual role in the formation of biomass heterosis of weichunbai No.3. Interactions between cis-regulatory and trans-regulatory variations can lead to gene expression changes in hybrids. If parental species have low genetic divergence, the relative frequency of trans-regulatory divergence surpasses that of cis-regulatory divergence, and trans-regulatory divergence accounts for a greater part of the regulatory divergence, resulting in additive inheritance (Zhang et al., 2019). This may explain why the majority of DEGs were additively expressed in this study.

**Figure 6:**
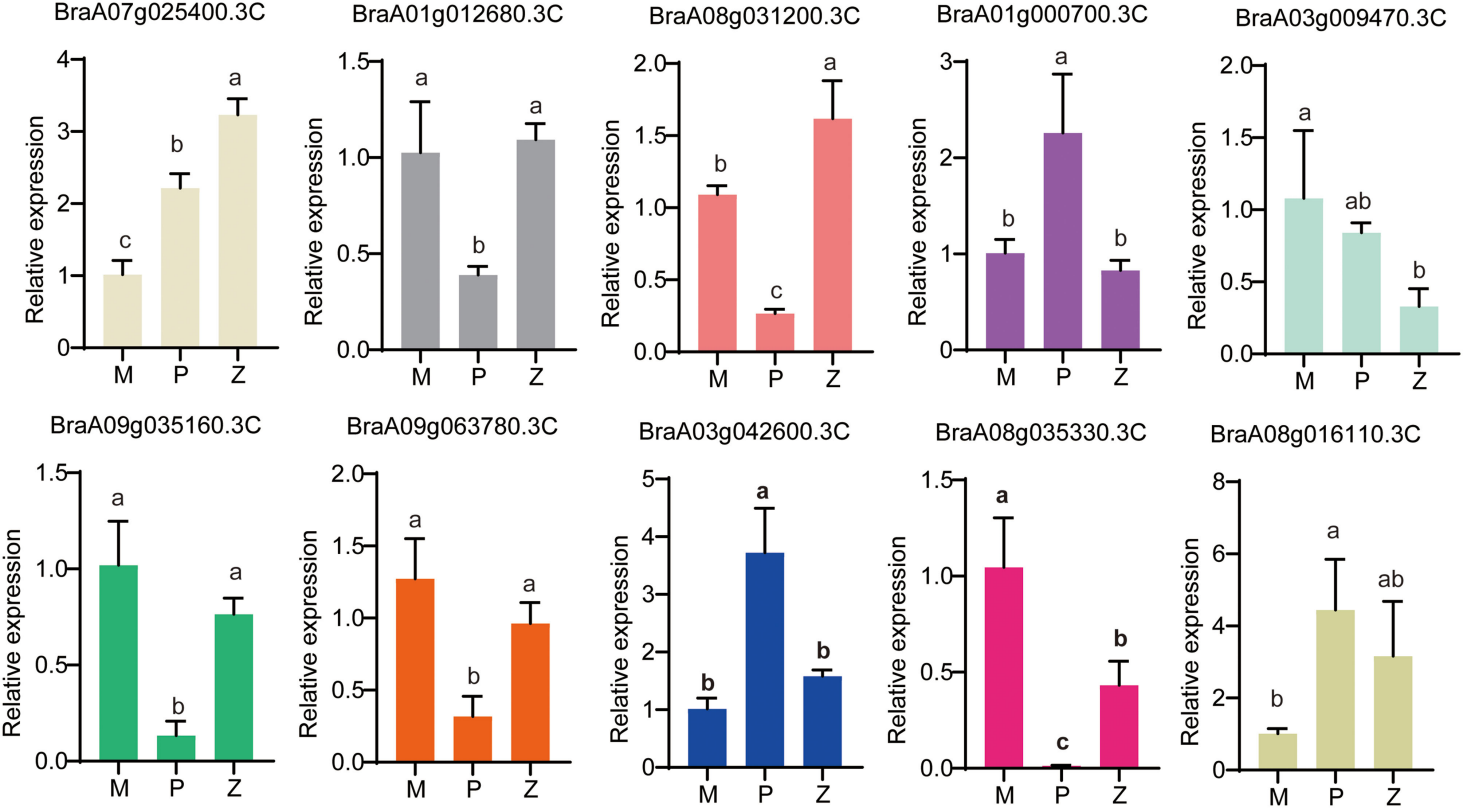
Validation of DEGs by qRT-PCR. The same letter indicates no significant difference (p > 0.05), and the different letter indicates significant difference (p< 0.05).

To demonstrate the accuracy and reproducibility of the RNA-seq data, ten DEGs were chosen for analysis of transcript abundance by qRT-PCR (**Figure 6**). BraA07g025400.3C showed the P1 type. BraA01g012680.3C and BraA08g031200.3C showed the P2 type. BraA09g035160.3C, BraA09g063780.3C, and BraA08g035330.3C exhibited the P3 type. BraA03g042600.3C and BraA08g016110.3C exhibited the P4 type. BraA01g000700.3C displayed the P5 type. BraA03g009470.3C displayed the P6 type. These results were consistent with those in the **Supplementary Table S4**. Therefore, the expression trends of these ten genes measured by qRT-PCR were in line with the RNA-seq results in the rosette stage, and the reliability of the RNA-seq data was confirmed.

### 3.4 Identification of the hub genes

**Figure 7:**
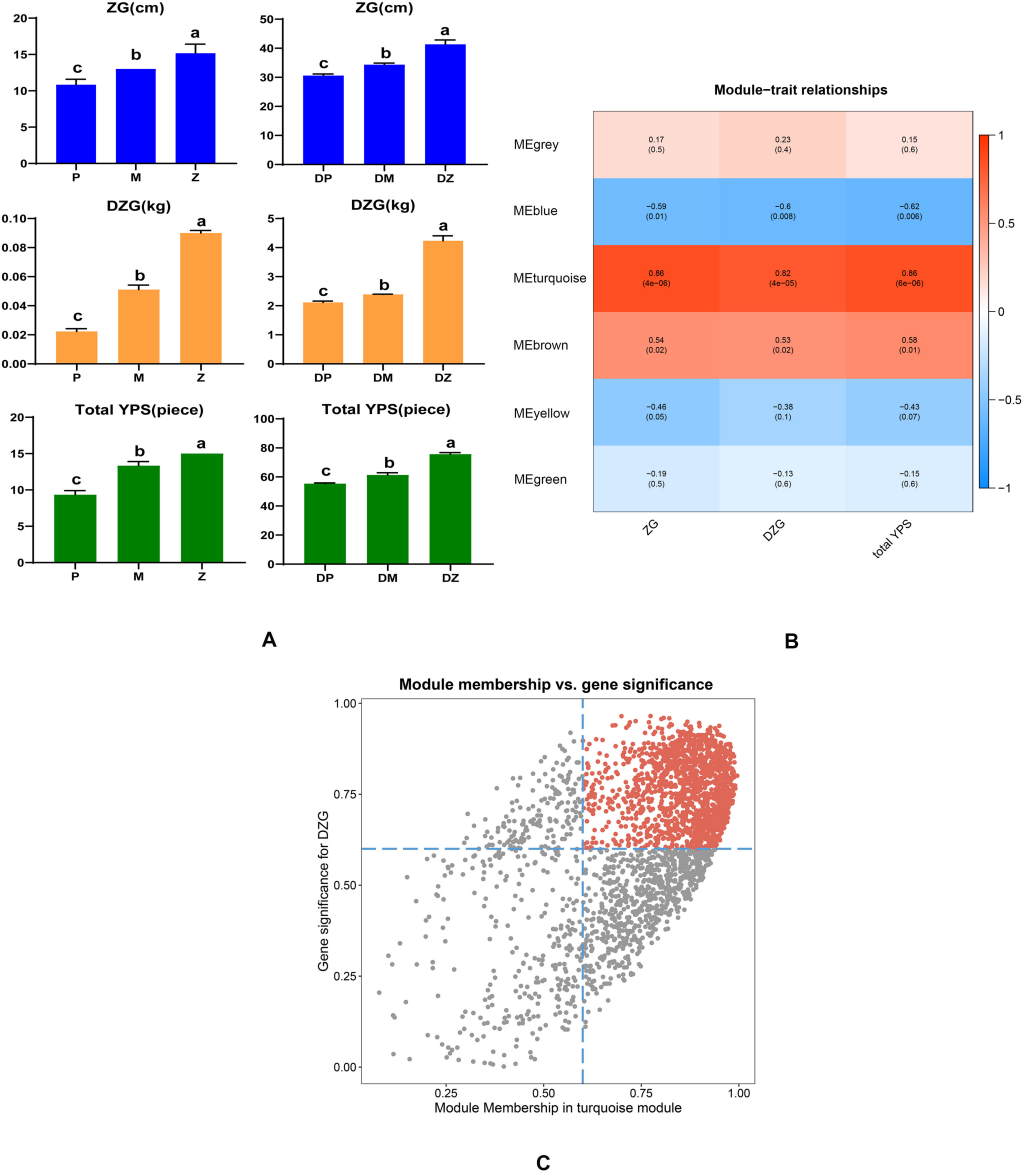
**(A)** Three growth characteristics of the hybrid and its parents in the rosette and mature stages. The same letter indicates no significant difference (p > 0.05), and the different letter indicates significant difference (p< 0.05). P, M and Z represent the paternal parent, the maternal parent and the hybrid in the rosette stage, respectively. DP, DM and DZ represent the paternal parent, the maternal parent and the hybrid in the mature stage, respectively. ZG represents the plant height, DZG represents the plant weight, and YPS represents the total leaf number. **(B)** Heatmap of the correlations between the three growth characteristics and module eigengenes according to WGCNA. **(C)** Scatter plots between gene significance for the DZG and module membership in the turquoise module. Each point corresponds to a gene in the turquoise module.

Three traits (plant height-ZG, plant weight-DZG and total leaf number-YPS) of the eighteen sequenced samples in the rosette and mature stages were measured and used for weighted gene coexpression network analysis. **Supplementary Table S8** showed that the average values of three horticultural traits of the Chinese cabbage hybrid and its parents. **Figure 7A** showed that there were differences in plant height, plant weight and total leaf number between the hybrid and its parents. In terms of plant weight, more obvious differences were detected between the hybrid and the two parents in the rosette and mature stages. Compared with its parents, the hybrid had a significant weight advantage. To analyze the relationships between DEGs and growth characteristics, WGCNA was applied to identify the gene modules highly related to growth characteristics. A total of six modules, including MEblue (1062 DEGs), MEbrown (642 DEGs), MEgreen (315 DEGs), MEgrey (30 DEGs), MEturquoise (2679 DEGs), and MEyellow (627 DEGs), were obtained (**Figure 7B**). When the correlation coefficient was r > 0.5 and p< 0.05, the modules and growth characteristics were considered significantly positively correlated. **Figure 7B** showed that the MEturquoise module was highly positively correlated with all growth characteristics (r > 0.8). The plant weight was our key concern; therefore, hub genes related to the plant weight were identified based on the cutoff thresholds: GS > 0.60 and MM> 0.60. A total of 1142 hub genes were confirmed in the MEturquoise module (**Figure 7C**; **Supplementary Table S6**). These 1142 genes were then subjected to KEGG analysis, focusing on the metabolic pathways related to photosynthesis. These genes were enriched into 98 metabolic pathways, two of which were related to photosynthesis, namely ‘photosynthesis - antenna proteins’ and ‘carbon fixation in photosynthetic organisms’ (**Supplementary Table S7**). One gene (BraA09g035160.3C) was enriched in ‘photosynthesis - antenna proteins’, and three genes (BraA03g004740.3C, BraA04g025990.3C, BraA08g029330.3C) were enriched in ‘carbon fixation in photosynthetic organisms’. The three genes (BraA03g004740.3C, BraA04g025990.3C, BraA08g029330.3C) had the lower expression levels and fold changes between the hybrid and its parents, while the BraA09g035160.3C gene presented a relatively high expression level and fold difference, and it was also a common DEG for the two stages. In addition, BraA09g035160.3C is an *LHCB1* gene associated with PS II and takes part in the light reaction during photosynthesis. Therefore, BraA09g035160.3C, which is related to photosynthesis, was selected for subsequent VIGS experiments.

### 3.5 Functional analysis of the BraA09g035160.3C gene

**Figure 8:**
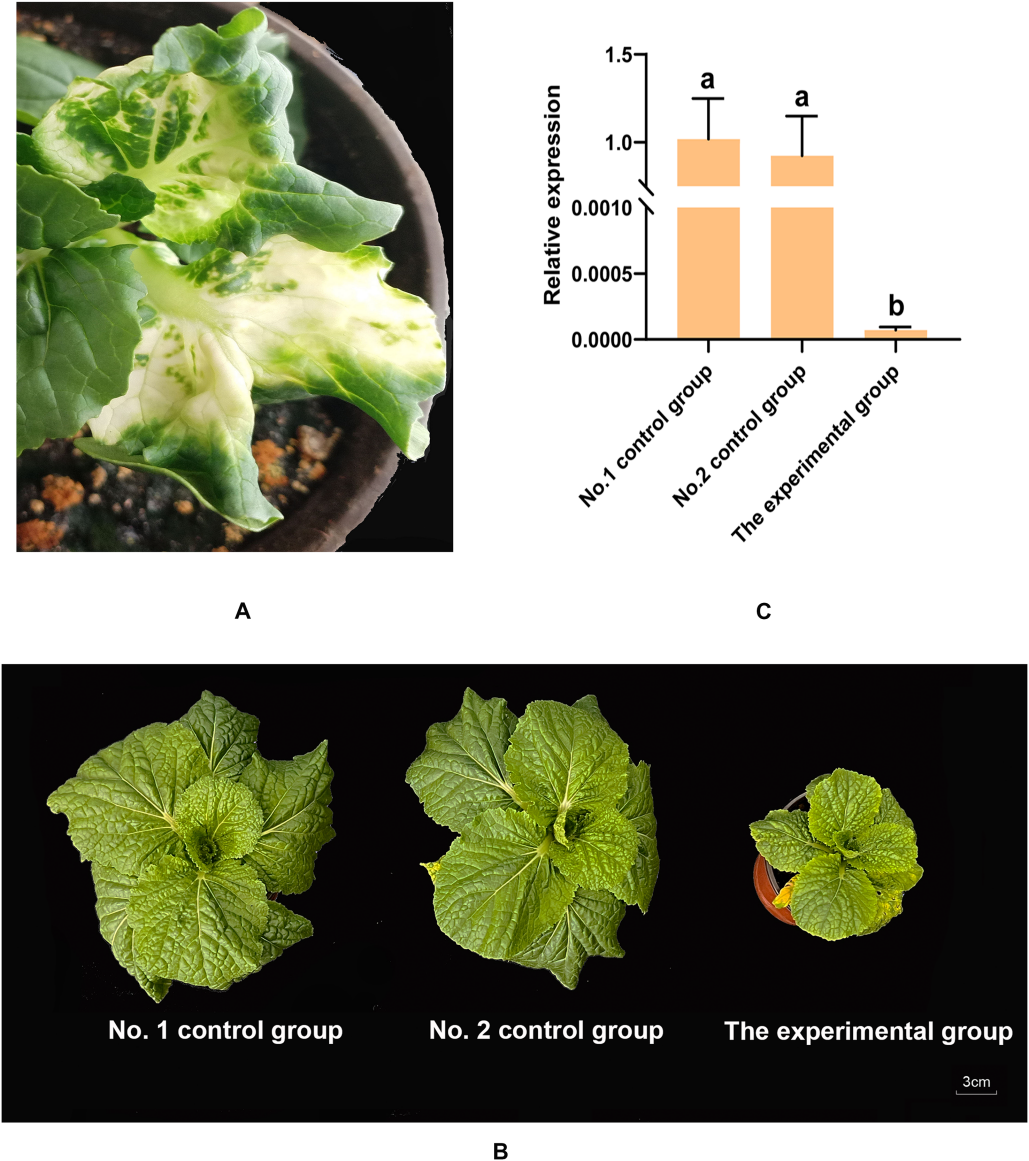
**(A)** White leaf phenotypes of *PDS*-silenced plant. **(B)** Plants in the two control groups and the experimental group at six weeks postinfiltration. **(C)** Relative expression levels of BraA09g035160.3C in the two control groups and the experimental group.

According to the previous methods (Yu et al., 2018), two *PDS* genes (BraA04g007910.3C and BraA08g023090.3C) were cloned and sequenced. Based on the results of multiple sequence alignment, a conserved 500 bp fragment was selected to construct a PCVA-*PDS* vector (**Supplementary Data**). At approximately 12 days postinfiltration, the *PDS*-silenced plants presented white leaves (**Figure 8A**), which demonstrated that the VIGS system used in this experiment functioned normally and correctly. To investigate the function of the BraA09g035160.3C gene, a loss-of-function study of the BraA09g035160.3C gene was also performed using VIGS technology. The full-length CDS of the BraA09g035160.3C gene was obtained by DNA sequencing. A 500 bp fragment was selected from the full-length CDS (**Supplementary Data**) to be cloned and inserted into the PCVA vector. The primers used for the VIGS and qRT–PCR experiments were listed in **Supplementary Table S9**. At approximately 12 days postinfiltration, there was no obvious difference in plant growth between the two control groups, whereas several smaller plants were observed in the experimental group. At approximately 6 weeks postinfiltration, significant differences in growth were observed between the experimental and control groups (**Figure 8B**). RNA was extracted from the leaves of the smaller plants in the experimental group and the leaves of the two control groups for subsequent experiments. The expression levels of the BraA09g035160.3C gene in the experimental group (slow-growing plants) and control groups were detected by qRT–PCR. The results revealed that there was no difference in the expression levels of the BraA09g035160.3C gene between the two control groups; compared with those in the two control groups, the relative expression levels of the BraA09g035160.3C were significantly lower in the slow-growing plants (**Figure 8C**). A significant decrease in the expression level of the BraA09g035160.3C gene led to slow plant growth, and this gene could affect plant growth by influencing photosynthesis efficiency. In conclusion, the expression of the BraA09g035160.3C gene could promote the growth of weichunbai No. 3 and improved leaf quality, contributing to the yield advantage formation of weichunbai No. 3.

## 4 Discussion

The expression levels of some genes in the plant hybrids change significantly, which causes alterations in various physiological and biochemical processes, resulting in changes in traits and the formation of dominant phenotypes. These genes are defined as DEGs (Fujimoto et al., 2018). DEGs in the plant hybrids can present multiple expression patterns. The DEGs between the wheat hybrid and its homozygous parents are classified into 12 patterns in seedling and spike tissues (Liu et al., 2018). The DEGs between the radish hybrid and its parents are also classified into 12 groups in the taproot tissue (Zhang et al., 2023). In this study, based on expression level comparisons of the DEGs among the hybrid and its parents, the DEGs in the rosette and mature stages were classified into eight and nine expression patterns, respectively. P4 (P>Z>M), with the most DEGs, was the dominant expression pattern, followed by P3 (M>Z>P) in the two stages. The DEGs in P3 and P4 accounted for 57.28% and 75.29% of the total DEGs in the rosette and mature stages, respectively. This result illustrated that the expression levels of the DEGs in the hybrid tended to be between those of the maternal parent and the paternal parent, with a greater tendency in the mature stage. Complicated regulatory mechanisms are the basis for changes in gene expression in hybrids. The reasons for changes in gene expression in hybrids are as follows: allelic sequence variation (Zhang et al., 2008), alterations in epigenetic modifications (Ni et al., 2009), and cis- and trans-regulatory variations (Combes et al., 2015). Although both cis- and trans-regulatory variations induce gene expression changes in cabbage head hybrids, cis-regulatory variations mediate most of the gene expression changes in these hybrids (Li et al., 2021a).

The increase in the expression levels of the photosynthesis-related genes can enhance the photosynthesis efficiency and the accumulation of hybrid biomass, promoting the formation of yield advantages (Fujimoto et al., 2018). Compared with those of its parents, the expression levels of key genes (e.g., those encoding phosphoenolpyruvate carboxylase and ribulose bisphosphate carboxylase) associated with the carbon fixation pathway of photosynthesis are significantly upregulated in superhybrid rice, the carbon fixation capacity is enhanced, and increased photosynthetic efficiency is subsequently detected, which is closely correlated with yield heterosis in superhybrid rice (Song et al., 2010). When Li et al. (2020) studied changes in protein and metabolite contents in maize hybrids, they reported that, compared with those of their parents, the contents of several key enzymes (e.g., malic enzyme, Rubisco activation, and transketolase) in the photosynthesis pathways of the hybrids were significantly increased, improving the photosynthesis efficiency of hybrids and promoting increases in the contents of various products (e.g., malate, fructose, and sucrose), contributing to the formation of yield advantages in hybrids. In addition, photosynthesis-related genes have also shown obviously upregulated expression trends in hybrids of cotton, wheat, and *Brassica napus* crops, which are beneficial for biomass accumulation in hybrids and promoting the formation of yield advantages (Liu et al., 2018; Zhu et al., 2020; Ding et al., 2021).

Leaves are the main edible organ of Chinese cabbage, and their size and quality directly affect the yield of Chinese cabbage hybrids. The ability of plants to photosynthesize in leaves has an important effect on plant weight. Therefore, changes in the photosynthesis process may affect the yield of Chinese cabbage hybrids. In this study, through WGCNA, a hub gene related to photosynthesis, BraA09g035160.3C, was identified, and its functional annotation revealed that it was an *LHCB1* gene. The light-harvesting antenna of PS II is composed of six different proteins, including three minor proteins encoded by the *LHCB4*, *LHCB5* and *LHCB6* genes and three proteins that make up the complex LHC II encoded by the *LHCB1*, *LHCB2* and *LHCB3* genes, of which the LHCB1 and LHCB2 proteins are the most abundant (Andersson et al., 2003). Plants with silenced expression of *LHCB1* and *LHCB2* present a pale green phenotype and low chlorophyll content (Andersson et al., 2003). LHCB1, as a light-absorbing antenna protein, absorbs light energy and subsequently transfers it to the photosynthesis reaction center in PS II. Compared with its parents, the LHCB1 protein is obviously more abundant due to the increase in the *LHCB1* gene expression level in a high-yielding wheat hybrid, which promotes light energy absorption and conversion, improving photosynthetic efficiency (Liu et al., 2020a). BraA09g035160.3C was significantly upregulated in the Chinese cabbage hybrid compared with its paternal parent. Through VIGS experiments, the expression level of the BraA09g035160.3C gene was significantly reduced in the Chinese cabbage hybrid, which could reduce the photosynthetic efficiency, thereby slowing hybrid growth. In conclusion, BraA09g035160.3C could play a vital role in the yield advantage formation of the Chinese cabbage hybrid.

For Chinese cabbage, leaf photosynthesis capacity is vital to the formation of the yield heterosis. In the F_1_ Chinese cabbage hybrid ‘Xin No. 3’, the expression levels of differentially expressed genes (DEGs) related to photosynthesis and chlorophyll synthesis are significantly different between the F_1_ hybrid and the parental lines, which results in enhanced photosynthetic capacity and chlorophyll content in the hybrid; furthermore, the rate of photosynthesis per unit area in the hybrid is higher than that in its parents from the seedling stage to the heading stage (Li et al., 2021b). During the cotyledon period of the Chinese cabbage hybrid, chloroplast-targeted genes, particularly those involved in photosynthesis (such as *LHCB* and *PSA*), exhibit higher expression levels in the F_1_ hybrid than in the parental lines do; the increased photosynthetic activity during the first week of cotyledon growth is crucial for increasing leaf size in F_1_ hybrids, even beyond the cotyledon stage (Saeki et al., 2016). The light reaction of photosynthesis occurs in chloroplast grana; therefore, increasing the number of grana is conducive to the photosynthesis of Chinese cabbage and has a positive effect on the Chinese cabbage yield (Yang, 2010). The number of grana thylakoids is positively associated with the expression level of the *LHCB1* gene, and the upregulation of the *LHCB1* gene can increase the number of grana thylakoids in pak choi hybrids (Liu et al., 2020b). Therefore, higher expression of the BraA09g035160.3C gene may increase the number of grana thylakoids in the weichunbai No. 3 hybrid, which contributed to the photosynthesis process and had a positive effect on the yield advantage of weichunbai No. 3. In addition to directly affecting the photosynthesis process through light reaction, the *LHCB1* gene can also indirectly regulate photosynthesis by affecting the number of chloroplast grana.

In-depth exploration of the reasons for the changes in the photosynthetic capacity of Chinese cabbage hybrids will help to elucidate the reasons for the formation of yield advantages of Chinese cabbage hybrids at the molecular level. During the breeding process of Chinese cabbage, genetic engineering technology can be used to alter the expression levels of genes related to photosynthesis, thereby improving the photosynthesis efficiency of Chinese cabbage and helping to increase the yield of high-quality Chinese cabbage.

## 5 Conclusion

In summary, a total of 5355 DEGs were identified between weichunbai No. 3 and its parents in the rosette and mature stages, and these DEGs exhibited diverse expression patterns among weichunbai No. 3 and its parents. In addition, the expression levels of the most differentially expressed genes in weichunbai No. 3 were higher than one of the parents but lower than another. The significant decrease in the expression level of BraA09g035160.3C, which is related to photosynthesis, led to slow plant growth and strongly affected the weight of weichunbai No. 3, indicating that BraA09g035160.3C could play an important regulatory role in the yield advantage formation of weichunbai No. 3. This study provides important clues for further exploration of the molecular mechanism of the yield advantage formation of Chinese cabbage hybrids.

## Data Availability

The datasets generated during the current study are available in the Sequence Read Archive (SRA) in NCBI (PRJNA1154826).
