# Supplementary material for: Unraveling yield heterosis in Chinese cabbage hybrid by comparative transcriptomic analysis and LHCB1 gene function analysis
Source: Front Plant Sci. 2025 Jul 2;16:1627259. doi: 10.3389/fpls.2025.1627259 (PMC12269066; doi:10.3389/fpls.2025.1627259)
Supplement: Supplementary file 1 [file DataSheet1.pdf]

Supplementary Material

Supplementary Data

The entire sequence of BraA09g035160.3C gene, and the conserved 500 bp fragments of *PDS* and BraA09g035160.3C genes.

> PDS-500 bp

TGCTGGTCCTTTGCAGGTAGTGTGTGTGGATATACCAAGGCCAGAGCTA
GAGAACACTGTCAATTTCTTGGAAGCTGCAAGTTTGTCTGCATCTTTCC
GTAGTGCTCCTCGTCCTGCAAAGCCTTTAAAAGTTGTCATTGCTGGTGC
TGGATTGGCTGGGCTGTCAACTGCAAAGTACCTGGCCGATGCAGGCCA
CCAACCTCTCTTGCTCAAAGCAAGAGATGTTCTTGGTGGAAAGATAGCT
GCATGGAAGGATGAAGATGGAGATTGGTATGAAACCGGTTTACATATAT
TTTTCGGTGCTTATCCGAACGTGCAGAACTTATTTGGAGAACTTGGGAT
TAATGATCGGTTGCAATGGAAGGAACACTCCATGATATTCGCCATGCCA
AGTAAACCTGGAGAATTTAGTAGATTTGATTTCCCAGATGTTCTACCAGC
ACCCTTAAACGGTATTTGGGCAATTTTGAGGAACAACGAGATGCTGACA
TGGCCAGAGA

> BraA09g035160.3C

ATGGCCTCTTCAACAATGGCTCTCTCCTCCCCTGCCTTCGCCGGAAAGGCCGTGAAGCTT
TCTCCTGCAGCATCAGAAGTCCTTGGAAGCGGCCGTGTGACAATGAGGAAGACCGTCGC
CAAGCCAAAGGGACCATCAGGCAGCCCATGGTACGGTTCCGAAAGAGTCAAGTACTTG
GGTCCATTCTCAGGCGAGCCACCGAGCTACCTTACCGGAGAGTTCCCAGGAGACTACGG
ATGGGACACCGCCGGCCTCTCAGCCGATCCCGAGACATTCGCAAGGAACCGTGAGCTAG
AAGTTATCCACTGCAGATGGGCCATGCTTGGAGCCCTAGGCTGTGTCTTCCCGGAGTTGT
TGGCCAGGAACGGAGTCAAGTTCGGAGAGGCGGTTTGGTTCAAGGCCGGTTCACAGATC
TTCAGCGAAGGAGGACTTGACTACTTGGGCAACCCGAGCTTGGTCCACGCTCAGAGCAT
CTTAGCTATTTGGGCCACTCAGGTGATCCTCATGGGAGCTGTAGAGGGTTACAGAGTCG
CCGGAGAGGGACCATTGGGAGAAGCAGAGGACTTGCTTTACCCAGGTGGCAGCTTCGAC
CCATTGGGTCTTGCTACCGACCCAGAGGCTTTCGCCGAGTTGAAGGTGAAGGAAATCAA
GAACGGAAGATTGGCTATGTTCTCTATGTTTGGATTCTTTGTTCAGGCCATTGTCACAGG
TAAGGGACCATTGGAGAACCTTGCTGACCATTTGGCTGATCCAGTCAACAACAATGCTT
GGGCCTTCGCAACCAACTTCGTTCCCGGAAAGTGA

> BraA09g035160.3C-500bp

ATGGCCTCTTCAACAATGGCTCTCTCCTCCCCTGCCTTCGCCGGAAAGG
CCGTGAAGCTTTCTCCTGCAGCATCAGAAGTCCTTGGAAGCGGCCGTG
TGACAATGAGGAAGACCGTCGCCAAGCCAAAGGGACCATCAGGCAGC
CCATGGTACGGTTCCGAAAGAGTCAAGTACTTGGGTCCATTCTCAGGCG
AGCCACCGAGCTACCTTACCGGAGAGTTCCCAGGAGACTACGGATGGG
ACACCGCCGGCCTCTCAGCCGATCCCGAGACATTCGCAAGGAACCGTG

AGCTAGAAGTTATCCACTGCAGATGGGCCATGCTTGGAGCCCTAGGCTG
TGTCTTCCCGGAGTTGTTGGCCAGGAACGGAGTCAAGTTCGGAGAGGC
GGTTTGGTTCAAGGCCGGTTCACAGATCTTCAGCGAAGGAGGACTTGA
CTACTTGGGCAACCCGAGCTTGGTCCACGCTCAGAGCATCTTAGCTATT
TGGGCCACTCAGGTGAT
